# Genetic characterization of *Mycobacterium tuberculosis* in the West Bank, Palestinian Territories

**DOI:** 10.1186/1756-0500-5-270

**Published:** 2012-06-07

**Authors:** Suheir Ereqat, Abedelmajeed Nasereddin, Kifaya Azmi, Ziad Abdeen, Charles L Greenblatt, Mark Spigelman, Nalin Rastogi, Gila Kahila Bar-Gal

**Affiliations:** 1 Al-Quds Nutrition and Health Research Institute, Faculty of Medicine, Al-Quds University, P. O. Box: 201760, Abu-Deis, Palestine; 2 Department of Microbiology and Molecular Genetics, The Hebrew University-Hadassah Medical School, Jerusalem, Israel; 3 Royal Free & University College Medical School, Centre for Clinical Microbiology, Royal Free Hospital, Rowland Hill Street, London, UK; 4 WHO Supranational TB Reference Laboratory, Unité de la Tuberculose et des Mycobactéries, Institut Pasteur de Guadeloupe, Abymes, Guadeloupe, France; 5 Koret School of Veterinary Medicine, The Robert H. Smith faculty of Agriculture, Food and Environment, The Hebrew University of Jerusalem, Rehovot, 76100, Israel

**Keywords:** Tuberculosis, Mycobacterium, Spoligotyping, MIRU, Genotyping

## Abstract

**Background:**

The World Health Organization (WHO) declared human tuberculosis (TB) a global health emergency and launched the “Global Plan to Stop Tuberculosis” which aims to save a million lives by 2015. Global control of TB is increasingly dependent on rapid and accurate genetic typing of species of the *Mycobacterium tuberculosis* (MTB) complex including *M. tuberculosis.* The aim of this study was to identify and genetically characterize the MTB isolates circulating in the West Bank, Palestinian Territories. Genotyping of the MTB isolates from patients with pulmonary TB was carried out using two molecular genetic techniques, spoligotyping and mycobacterial interspersed repetitive units-variable number of tandem repeat (MIRU-VNTR) supported by analysis of the MTB specific deletion 1 (TbD1).

**Findings:**

A total of 17 MTB patterns were obtained from the 31 clinical isolates analyzed by spoligotyping; corresponding to 2 orphans and 15 shared-types (SITs). Fourteen SITs matched a preexisting shared-type in the SITVIT2 database, whereas a single shared-type SIT3348 was newly created. The most common spoligotyping profile was SIT53 (T1 variant), identified in 35.5 % of the TB cases studied. Genetic characterization of 22 clinical isolates via the 15 loci MIRU-VNTR typing distinguished 19 patterns. The 15-loci MIT144 and MIT145 were newly created within this study. Both methods determined the present of *M. bovis* strains among the isolates.

**Conclusions:**

Significant diversity among the MTB isolates circulating in the West Bank was identified with SIT53-T1 genotype being the most frequent strain. Our results are used as reference database of the strains circulating in our region and may facilitate the implementation of an efficient TB control program.

## Findings

### Background

Tuberculosis (TB) caused by *Mycobacterium tuberculosis* (MTB) complex persists as one of the most important public health problems worldwide, despite the global efforts to control and eradicate this disease. The World Health Organization (WHO) has declared that TB is a global epidemic with high morbidity and mortality, especially in low-income countries. Important progress has been made in the last decade in TB diagnostics and control programs (http://www.who.int/tb/laboratory/en/). Rapid detection and adequate therapy to prevent MTB transmission are key factors in TB control programs. In the last years, molecular typing approaches have greatly enhanced our understanding of TB epidemiology by indicating possible epidemiological links between TB patients and by detecting suspected outbreaks [1]. Spoligotyping, an identification method based on the direct repeat (DR) region pattern, allows simultaneous strain differentiation of MTB in clinical specimens without the need for culture [2]. The clinical usefulness of spoligotyping is determined by its rapidity, both in detecting causative TB pathogens and in providing epidemiologic information on strain identities. The mycobacterial interspersed repetitive units-variable number of tandem repeats (MIRU-VNTR) typing has been shown to be a reliable and reproducible method with high discriminatory power to distinguish between MTB strains [1]. A combination of the two methods has been used to replace typing via restriction fragment length polymorphisms based on the insertion sequence *IS6110*. Importantly, some mutations which have been described in MTB genes that are associated with resistance to rifampin (RIF) or isonizide (INH) were found to be specific to particular strain families [3].

In the West Bank, with a population of approximately 2.44 million inhabitants, TB incidence rate is low. This can be explained as representing the precise incidence of the disease or a result of a misdiagnosis due to the low sensitivity of the diagnostic methods which are primarily based on microscopic examination of self-expectorated sputum stained for acid fast bacilli. Several cases of multi drug resistance (MDR) and non-compliance to therapy were reported in the last few years [4,5]. This recognized that the deterioration in the economical, nutritional and health situations caused by regional conflict increases the likelihood of an increase in misdiagnosed patients and the rise of MDR TB case. The TB diagnostic criteria are well established and practiced in the Palestinian Territories. Pulmonary TB is suspected among patients that cough for more than 2 weeks and do not respond to nonspecific antibiotics. These patients are referred to the central laboratory at the Palestinian Ministry of Health for microbiological testing. Three sputum samples are collected from each patient for microscopic examination and culture using the conventional Lowenstein-Jensen (L-J) media. Although conventional microbiology still constitutes the principal tool for the diagnosis of TB and appropriate for laboratories with minimal infrastructure, direct microscopy testing has low sensitivity [5], and culture requires a long period of time, leading to significant delays in diagnosis and treatment, which gives rise to development of drug resistance [6]. Therefore, early diagnosis, effective treatment and identifying factors influencing disease dynamics are major features in the control of TB in our region. A detailed genetic study of the regional MTB isolates, identification of the genotypes associated with drug resistance and specifically evaluation of the appearance of new MDR-TB strains in the studied population can facilitate both a more effective drug therapy regime and give information at the molecular epidemiological level. The goal of our study was to identify and molecularly characterize the MTB strains circulating in the Palestinian Territories for further TB management programs.

### Methods

#### Patient’s characteristics and clinical specimens

**Figure 1:**
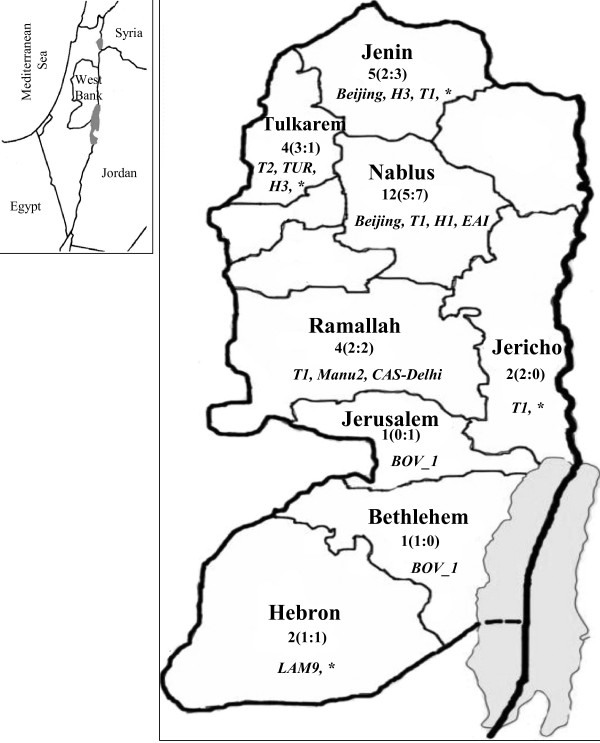
**Distribution of*Mycobacterium tuberculosis*strains inthe West Bank, Palestinian Territories during 2005–2010.** The MTB strains, defined by spoligotyping, are listed in each district. * = Unknown MTB lineages. Number of patients studied and the sex ratios [males:females] are included per district.

Overall 53 DNA samples were studied: 22 clinical isolates that were retrieved from sputum samples and 31-Ziehl Nelseen (ZN) stained sputum smears collected during 2005–2010. All isolates were identified as MTB, except of two that were *M. bovis*[7]. Smears and their corresponding isolates were retrieved from 31 pulmonary TB patients originating from 8 different districts across the Palestinian Territories (Figure1) and were diagnosed on the basis of clinical symptoms, chest X ray and bacteriological examination. All the patients were diagnosed as TB positive based on the smear test. Among them, 9 patients were under TB therapy at the time of sampling and were found culture negative. Twenty-nine patients were adults (18-71 years) and two were children (2 and 4 years old). Out of the 31 patients, 16 were males and 15 were females giving a sex ratio of 1.1:1. Of all cases, eight patients (26 %) had newly diagnosed TB in 2010. It is important to note that although the sample size is relatively small, compared to other TB studies, it comprised the largest sample available in the last 5 years in the West Bank including all the smear positive cases of pulmonary TB that were reported by the Palestinian ministry of health in 2009–2010 (n = 11).

Since this study involved analysis of archival samples and clinical isolates obtained during routine diagnostic work, patients were not asked to give their informed consent. However, Al-Quds University ethics committee approved the study protocol.

#### Spoligotyping

All samples (22 clinical isolates and 31 ZN- stained smears) were analyzed by spoligotyping as previously described [2]. The direct repeat (DR) region was amplified using DRa and DRb primers. The amplified biotinylated products hybridized to a set of 43 oligonucleotides covalently bound to a membrane (Isogen Life Science B.V., Utrecht, The Netherlands). The hybridized PCR products were then incubated with streptavidin-peroxidase conjugate. The membrane was incubated in chemoluminescence substrate (Amersham, Little Chalfont, England), then it was exposed to X-ray film (Hyperfilm ECL, Amersham) according to the manufacturer’s instruction. For reproducibility and specificity of the spoligotyping; duplicate samples (the slides and their corresponding isolates) together with known strains (*H37Rv* and *M. bovis BCG*) were tested.

#### Analysis of *M. tuberculosis* specific deletion 1 (TbD1)

The presence or absence of MTB specific deletion (TbD1) was analyzed by PCR as previously described [8]. Two PCR’s were performed for each sample using either primers complementary to the internal sequences of the TbD1 region or primers complementary to the sequences flanking the deleted region.

#### Mycobacterial interspersed repetitive units-variable number of tandem repeat (MIRU-VNTR)

The 15-loci MIRU-VNTR typing was conducted as described previously [1]. Several attempts to apply the 15-loci MIRU-VNTR typing on DNA extracts obtained from ZN-stained slides failed and thus it was applied only on DNA samples obtained from cultured bacteria (n = 22). For three relapse patients, an additional seven isolates were analyzed to replicate and further support the results. Each locus was amplified individually. The PCR fragments were separated by electrophoresis using 2 % NuSieve gel. The fragments size was estimated by comparison with 50 bp and 100 bp DNA molecular weight ladders and was independently checked visually by two investigators. The table used for MIRU-VNTR allele scoring is available online in the technical guide ((supply 2005), http://www.miru-vntrplus.org/MIRU/index.faces).

#### Genetic detection of the *KatG* S315T mutation by direct sequencing

All samples (n = 31) were analyzed for the presence of *KatG* S315T mutation that is known to be associated with INH resistance. A partial gene sequence of the *katG* gene targeting the G to C substitution at codon 315 was amplified and sequenced as previously described [9].

### Results

#### Spoligotyping and TbD1 region

Spoligotyping was used to characterize the strain of all samples (n = 31). In addition, all samples were tested for presence of the TbD1 region, and as expected, this region was identified in two *M. bovis* strains. Interestingly, one MTB isolate (477777277413771) also harbored the TbD1 region, which was further characterized by presence of DR spacer 33 and absence of spacers 29–32 and 34 (Table 1).

**Table 1:** **Description of spoligotyping defined lineages/sublineages among*M. tuberculosis*strains isolated from patients residing in the West Bank/Palestinian territories, their proportion in study versus SITVIT2 database**

**SIT (Lineage) Octal Number Spoligotype Description**	**Number (%) in study**	**% in study vs. SITVIT2 database**
Orphan (T1) 037637037760771	1 (3.23)	100
□□□□■■■■■■■□□■■■■■□□□□■■■■■■■■■■□□□□■■■■■■■		
Orphan (T1) 777777377660771	1 (3.23)	100
■■■■■■■■■■■■■■■■■■□■■■■■■■■■■□■■□□□□■■■■■■■		
SIT1 (Beijing) 000000000003771	2 (6.45)	0.02
□□□□□□□□□□□□□□□□□□□□□□□□□□□□□□□□□□■■■■■■■■■		
SIT4 (Unk) 000000007760771	3 (9.68)	0.93
□□□□□□□□□□□□□□□□□□□□□□□□■■■■■■■■□□□□■■■■■■■		
SIT10 (EAI8-MDG) 477777277413771	1 (3.23)	1.25
■□□■■■■■■■■■■■■■■■□■□■■■■■■■□□□□■□■■■■■■■■■		
SIT26 (CAS1-Delhi) 703777740003771	1 (3.23)	0.09
■■■□□□□■■■■■■■■■■■■■■■□□□□□□□□□□□□■■■■■■■■■		
SIT42 (LAM9)777777607760771	1 (3.23)	0.03
■■■■■■■■■■■■■■■■■■■■□□□□■■■■■■■■□□□□■■■■■■■		
SIT52 (T2) 777777777760731	1 (3.23)	0.13
■■■■■■■■■■■■■■■■■■■■■■■■■■■■■■■■□□□□■■■□■■■		
SIT53 (T1) 777777777760771	11 (35.48)	0.2
■■■■■■■■■■■■■■■■■■■■■■■■■■■■■■■■□□□□■■■■■■■		
SIT54 (Manu2) 777777777763771	1 (3.23)	0.47
■■■■■■■■■■■■■■■■■■■■■■■■■■■■■■■■□□■■■■■■■■■		
SIT62 (H1) 777777774020731	1 (3.23)	0.2
■■■■■■■■■■■■■■■■■■■■■■■■■□□□□□□■□□□□■■■□■■■		
SIT118 (T1) 777767777760771	1 (3.23)	0.71
■■■■■■■■■■■■■■□■■■■■■■■■■■■■■■■■□□□□■■■■■■■		
SIT367 (TUR) 777737404760771	1 (3.23)	8.33
■■■■■■■■■■■■□■■■■■■□□□□□■□□■■■■■□□□□■■■■■■■		
SIT390 (H3) 777777777620771	1 (3.23)	3.45
■■■■■■■■■■■■■■■■■■■■■■■■■■■■■□□■□□□□■■■■■■■		
SIT482 (BOV_1) 676773777777600	2 (6.45)	0.27
■■□■■■■■□■■■■■■□■■■■■■■■■■■■■■■■■■■■■■□□□□□		
SIT750 (H3) 003777740003171	1 (3.23)	4.76
□□□□■■■■■■■□■■■■■■■□■□□□□□□□□□□■□□□□■■■■■■■		
SIT3348* (Unk) 003777740003171	1 (3.23)	50
□□□□□□□■■■■■■■■■■■■■■■□□□□□□□□□□□■■□□■■■■■■		

Note that SIT3348* was "newly created” within this study. Lineage designations were according to revised SpolDB4 rules [10]. Unk (unknown) designates patterns that did not belong to known lineages described in the database; TUR (Turkey) designates the newly assigned Turkey lineage, previously classified as LAM7-TUR [11].

The spoligotyping patterns obtained from ZN-stained slides were completely identical to those obtained from their corresponding culture isolates. For the 31 samples analyzed, a total of 17 different patterns were found (Table 1), of them, 13 isolates had unique patterns (they did not match with another strain within this study). Among these patterns, two isolates (777777377660771 and 037637037760771) were orphan types; they had no homologue in the SITVIT2 database. A single shared-type SIT3348 was "newly created" based on a single strain from this study that matched an orphan in the database from Pakistan (Table 1). The pattern obtained was classified as “unknown” in SITVIT2, even though the spoligotyping pattern of this strain resembles the EAI lineage (with the exception of absence of spacer 33, normally present in EAI). The TbD1 deletion found in the strain distinguished it from the EAI lineage. Lastly, based on expert-based visual interpretation, the two orphan strains were tentatively assigned to T1 sublineage and might result from microevolution of the circulating T lineage genotypes.

The other 18 isolates analyzed by spoligotyping were distributed in four clusters. The largest cluster (n = 11) displayed a typical T1 spoligotype (SIT53). The second cluster (n = 3) was the SIT4 genotype (previously assigned to LAM 3 and S/convergent; a careful examination showed that its pattern did not correspond to any well-defined lineages in SITVIT2, hence it was relabeled “unknown”). The third cluster contained two isolates of SIT1 belonging to the Beijing genotype while the fourth cluster (n = 2) belonged to SIT482 *M. bovis* (BOV_1 sublineage; Table 1).

#### MIRU-VNTR

**Figure 2:**
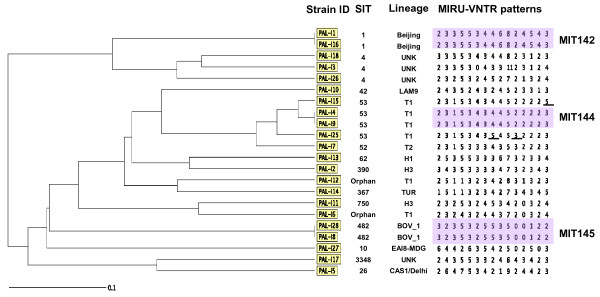
**Genetic relationships among the TB isolates as revealed by MIRU-VNTRs.** The online resource MIRU-VNTRplus (http://www.miru-vntrplus.org/MIRU/index.faces) was used for the phylogentic tree analysis. MIT: MIRU International Type. MIT144 and MIT145 were newly created with in this study. The underlined loci represented single and double locus variants of MIT144.

The 15-loci MIRU-VNTR typing results were available for 22 culture isolates. A total of 19 distinct patterns were acquired. Three clusters of two isolates each were obtained but the majority of isolates (n = 16) did not cluster (Figure2). The 15-loci MIT144 and 145 were newly created within this study, while the 15-loci MIT142 was already reported in four MTB clinical isolates in the database. No discrepancies were found between MIRU-VNTR classified patterns in multiple isolates from the same patient corresponding to relapses at different time points. Thus the second episodes of TB were probably due to reactivation of the indigenous strain revealing the stability and reproducibility of the MIRU-VNTR typing.

#### Genetic detection of drug resistance strains

In this study, the substitution known to be associated with INH resistance at codon 315 [S (AGC) to T (ACC)] was identified in the Beijing and LAM 9 isolates. One representative sequence was deposited in the GenBank (Accession no. JN411081). The mutation was not found in all of the other 28 isolates, representing various genotypes. Identification of the LAM 9 and Beijing isolates as drug resistance strains was further supported by our previous study that characterized the hot spot region of *rpoB* gene [5]. Two mutations (S < 531 > L and L < 572 > F) were found in the two Beijing strains and one double mutation (S < 531 > F) was identified in one strain belonged to the LAM 9 family that are known to be associated with drug resistance [5].

#### Synthesis of the characterization methods

Comparison of the results from the molecular characterization methods was conducted on 22 isolates. The 15-loci MIRU-VNTR analysis confirmed the clustering results obtained by the spoligotyping for only two clusters (SIT1 and SIT482*M. bovis*)*.* The two isolates (PAL-I1, PAL-I16) that were identified as Beijing strain, SIT1, via spoligotyping (Table 1) exhibited identical MIRU-VNTR patterns and were identified as MIT142 (Figure2). The mutations found in the *rpoB* and *KatG* genes supported the identification of these strains as MDR. The two patients, represented by these isolates, were not related to each other, they lived in two different districts, infected in different years (2007 and 2010) and were treated in different clinics. In addition, the two isolates that matched SIT482, *M. bovis* strain, had identical MIRU-VNTR patterns and were identified as MIT145 (Figure2). Moreover, the TbD1 region that characterizes *M. bovis* was identified in these isolates. These isolates were obtained from two cousins who were children, one of them was infected in 2006 and the other in 2010. Additional information that may help in understanding the transmission of the infection is unavailable.

However, the two clusters identified by spoligotyping as SIT4 and T1 family strains (SIT53) (Table 1) were not supported by the MIRU-VNTR analysis. The three isolates, identified as SIT4 by spoligotyping, were found to differ from each other and typed to three unique patterns by the MIRU-VNTR genotyping. These isolates were obtained from three unrelated patients who live in three separated districts, lacking any known epidemiological link.

The four isolates identified as T1 family strains (SIT53) via spoligotyping, were typed by MIRU-VNTR as MIT144 and variants of MIT144 (Figure2). Among them, two isolates (PAL-I4, PAL-I9) identified as MIT144 were obtained from two close contacts that were diagnosed and treated in the same clinic. The other two isolates (PAL-I15, PAL-I25) represented single and double locus variants of MIT144 (Figure2), without any clear epidemiological link between the patients.

### Discussion

In the present study, genetic analyses by spoligotyping and 15-loci MIRU-VNTR typing were determined to reveal the molecular genetic features of MTB strains circulating in the West Bank.

The predominant genotype found in the West Bank was that of SIT53-T1 (n = 11) known as an ubiquitous spoligotype. Our findings are consistent with other studies, which indicate that SIT53-T1 is the most frequent strain found in America, Europe, Asia and Africa. Although the T family is one of the most prevalent worldwide, it remains an ill-defined family of MTB*,* which needs to be further characterized [10].

The results obtained with spoligotyping in our study suggested a possible epidemiological link between three patients whose isolates displayed the same spoligotyping patterns (SIT4). However, when these isolates were typed by MIRU-VNTR, they showed different diverse patterns. The 15-loci MIRU-VNTR typing significantly reduced the number of epidemiological links among the studied isolates while spoligotyping overestimated these links. These differences between the two typing methods were found in other published studies [12,13] indicating that MIRU-VNTR typing is more powerful tool for molecular epidemiological studies of MTB. In several cases the spoligotyping and the MIRU-VNTR identified the same MTB strain: for example the identification of the Beijing (SIT1/MIT142) and *M. bovis* strains. Interestingly, all the 15-loci MIT142 strains (n = 6) in the SITVIT database belong to the Beijing lineage, i.e., SIT1/Beijing are represented by five cases, and SIT190/Beijing represented by one case.

We believe that using the 24-loci MIRU-VNTR, could further increase the discriminatory power of this method particularly for homogenous strains such as the *M. bovis* and Beijing strains [1,14]. Moreover, a more detailed identification of the strains will assist in determining the dynamic of the MTB strains in the West Bank especially introduction of new MTB strains in the region.

The study of theTbD1 region showed that the majority of isolates (n = 28) belonged to the TbD1 deleted modern strains, whereas only 3 strains (two *M. bovis* and one EAI strain) had TbD1 intact. However, all EAI strains that had the TbD1 region were designated ancestral MTB strains since they belong to a lineage that split from MTB strains before the deletion of TbD1 occurred [15]. Other interesting strains that were identified in this study: (i) one strain belonging to the Manu family; this strain was obtained from a non-local patient from Indonesia who visited the West Bank and was under treatment at the time of sampling; (ii) one strain of the CAS family (sample PAL-I5), which is thought to represent a possible ancestor of the Beijing family [10]; and (iii) strains from Haarlem (H1, H3), T2, TUR and LAM 9 families. Overall, 19 different strains (as revealed by MIRU-VNTR typing) were detected from various communities located in different areas in the West Bank. The different genotypes which were found in our region (Figure1), that are represented in many geographical regions worldwide, supports world globalization.

The Beijing and LAM 9 strains that were identified among the studied isolates were further found to carry the mutations along the *katG* and the *rpoB* genes known to be associated with drug resistance [5,9] and thus were considered as MDR-TB strains. It is important to note that these strains were obtained from three unrelated patients who did not respond to the first line of anti-tuberculosis drugs regimen. However, both genotypes (the Beijing and LAM) showed MDR phenotype association in other studies [16,17]. As drug susceptibility testing has not been applied yet in the West Bank, treatment failure and suspicious drug resistance cases are referred to neighboring countries for further investigations. Based on epidemiologic data, Beijing family strains may have a selective advantage over other MTB strains, enabling aggressive expanding among the population [18]. Therefore, the finding of Beijing strain in the Palestinian Territories underlines the emergence of Beijing genotype in the region, which constitutes a major public health risk in a vulnerable population and reinforcing the need to raise awareness of the disease in the region. Our results suggests that the genetic screening for the hot spot region of the *rpoB* and the S315T *katG* mutation may rapidly provide information for anti-TB regimen selection and possibly assist in tracing transmission of MDR strains.

### Conclusions

This study presents the first overview of *M. tuberculosis* genotypes circulating in the West Bank Palestinian Territories using two different molecular typing methods. In addition, it provides preliminary insight into the molecular diversity of the studied population including identification of mutations associated with the development of resistance to RIF and INH, belonging to a certain mycobacterial genotype.

The genotyping obtained in this study represents an early attempt to establish a database of strain types circulating in our region. This database will enable us to trace the relationships among strains, to restrict the chain of transmission between different communities, reveal the sources of infection and facilitate the implementation of effective TB control program. Further studies may provide a better understanding of the molecular epidemiology and biodiversity of *M. tuberculosis* in the region.

## Competing interests

The authors declare that they have no competing interests.

## Authors’ contributions

SE and AN carried out the experimental procedures and analyzed the data. SE wrote the manuscript and participated in the study design. KA participated in samples collection and DNA extraction. MS, CLG and NR analyzed the data, and proofread the manuscript. ZA and GKBG conceived and designed the study, provided the laboratory facilities, supervised the work and proofread the manuscript. All authors read and approved the final manuscript.
